# Supplementary material for: Post-acute COVID-19 associated with evidence of bystander T-cell activation and a recurring antibiotic-resistant bacterial pneumonia
Source: eLife. 2020 Dec 17;9:e63430. doi: 10.7554/eLife.63430 (PMC7775105; doi:10.7554/eLife.63430)
Supplement: Supplementary file 1. [file elife-63430-supp1.pdf]

Supplementary Table S1. Details of the antibodies used for the flow cytometry experiments.

Antibody	Company	clone	concentration used
Brilliant Violet 650™ anti-human CD4	Biolegend	RPA-T4	0.625:50
Brilliant Violet 605™ anti-human HLA-DR	Biolegend	L243	2.5:50
Brilliant Violet 711™ anti-human CD38	Biolegend	HIT2	2.5:50
Zombie Aqua™ Fixable Viability Kit	Biolegend		1:1000
Brilliant Violet 421™ anti-human Ki-67	Biolegend	Ki-67	3:50
PE-CF594 Mouse Anti-Human CD56	BD Biosciences	NCAM16.2	0.5:50
PE anti-human CD16	Biolegend	3G8	2.5:50
APC/Cyanine7 anti-human CD8	Biolegend	SK1	3:50
Alexa Fluor® 700 Mouse Anti-Human CD3	BD Biosciences	UCHT1	1:50
V450 Mouse Anti-Human IFN-γ	BD Biosciences	B27	3:50
Alexa Fluor® 488 anti-human CD107a (LAMP-1)	Biolegend	H4A3	2.25:150
PE/Dazzle™ 594 anti-human TNF-α	Biolegend	MAb11	3:50
Brilliant Violet 785™ anti-human CD163	Biolegend	GHI/61	2.5:50
Brilliant Violet 605™ anti-human CD14	Biolegend	M5E2	2.5:50
Alexa Fluor® 488 anti-human CD68	Biolegend	Y1/82A	5:50
PE/Dazzle™ 594 anti-human CD66b	Biolegend	G10F5	5:50
PE anti-human TCR γ/δ	Biolegend	B1	2.5:50
APC/Cyanine7 anti-human CD16	Biolegend	3G8	0.5:50
APC anti-human CD80	Biolegend	2D10	2.5:50
PerCP-Cy™5.5 Mouse Anti-Human CD3	BD Biosciences	UCHT1	2:50
